# Characterization of the In Situ Stress State of Blood Clots in Ischemic Stroke: The Effect of Initial Conditions and Arterial Interaction

**DOI:** 10.1002/cnm.70094

**Published:** 2025-10-03

**Authors:** Jose L. Monclova, Scott D. Simon, Keefe B. Manning, Francesco Costanzo

**Affiliations:** ^1^ Department of Biomedical Engineering The Pennsylvania State University University Park Pennsylvania USA; ^2^ Department of Neurosurgery Penn State College of Medicine Hershey Pennsylvania USA; ^3^ Department of Surgery Penn State College of Medicine Hershey Pennsylvania USA; ^4^ Department of Engineering Science and Mechanics The Pennsylvania State University University Park Pennsylvania USA

## Abstract

Ischemic stroke, caused by a blood clot lodging in cerebral vasculature, is a leading cause of death worldwide. The mechanics of vessel occlusion and the influence of residual stress on thrombectomy outcomes remain poorly understood. Most computational studies neglect arterial residual stress and the deformation a clot undergoes as it lodges, both of which elevate system stresses. Here, we introduce a method to simulate the initial state of a clot lodged in an idealized artery with residual stress. In this study, the artery is formulated as two concentric right cylinders with fibers embedded in an isotropic mesh, with a pre‐deformation used to incorporate residual stress. A base equilibrium state of an elastic clot is simulated in continuous contact with the arterial wall. The opening angle of the artery, un‐lodged‐to‐lodged dimensional ratios, and stiffness of the clot are varied in parametric sweeps to characterize the traction forces of the clot into the arterial wall. An aspiration pressure is applied to the proximal end of the clot to determine the pressures necessary to begin tensile detachment of the clot. As the artery opening angle increased, removal aspiration pressures increased, while the pressures decreased with increasing artery fiber orientation. The stress‐free‐to‐lodged length ratio of the clot influenced the removal aspiration pressure, with pressures increasing nearly a thousand‐fold with increased ratio. By incorporating different factors that contribute to the stress state of the system, this study provides a library of realistic initial conditions for simulating aspiration thrombectomy and validating new surgical techniques.

## Graphical abstract

Ischemic stroke is a leading cause of death worldwide, yet clot lodging mechanics remain poorly understood. Most *in silico* studies assume a stress‐free clot, though both the clot and artery are likely in a pre‐stressed state at the point of thrombectomy procedures. We provide realistic parameter ranges for clot dimensions and artery residual stresses as initial conditions for simulations. This study highlights the correlation between these conditions and aspiration thrombectomy outcomes, improving model accuracy and clinical relevance.

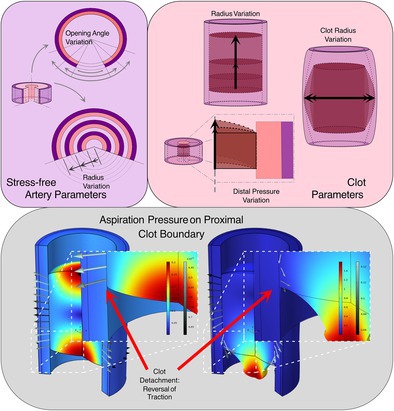

## 1 Introduction

What happens in the critical moments following a large cerebral vessel occlusion during an ischemic stroke? The sudden cessation of blood flow to vital regions of the brain can result in irreversible damage in minutes, and yet little is known about the biomechanics of this problem. Stroke is the third leading cause of death worldwide [1], with nearly 87% of all strokes resulting from vessel occlusion rather than vessel hemorrhage [2, 3]. Current treatment methods for acute ischemic stroke (AIS) include administration of a thrombolytic drug and mechanical thrombectomy, where an aspiration catheter or a stent retriever is used to mechanically dislodge the clot. Despite advances in current AIS treatment technology, nearly 15% of stroke cases result in some degree of morbidity or death [4, 5]. The underlying causes are a matter of investigation, but it is largely thought that the mechanical properties of the blood clots, coupled with the conditions experienced by the clot in the vessel, impact the outcomes of thrombectomy techniques (c.f., e.g., [6, 7, 8]). However, due to the sensitive anatomy involved and the critical nature of the procedures, large scale clinical studies cannot be performed to understand these complex interactions, highlighting the need for validated computational studies to understand these phenomena. Understanding the mechanics involved in the initial/boundary value problem (IBVP) is critical in determining the underlying causes in endovascular thrombectomy (EVT) procedures.

Recent studies have highlighted the role of the thrombus mechanical properties in recanalization rates for AIS surgeries, with clot composition, stiffness, and size correlating with surgical outcomes (c.f., e.g., [9, 10, 11]). Several studies, including those by Cahalane et al. [12], Good [13], Monclova et al. [14], and Boodt et al. [15], among others, found that thrombi with higher concentrations of red blood cells (RBCs) tended to be larger and less stiff than their platelet‐rich counterparts (c.f., e.g., [9, 16, 17]). Other groups found that despite stiffness differences, the size of the clot tended to play a larger role in recanalization outcomes [18, 19, 20]. Several studies have demonstrated that cardioembolic thrombi, which have been shown to be RBC‐rich, tended to increase the likelihood of incomplete clot removal [17, 21, 22]. Khismatullin et al. [23, 24, 25] demonstrated a contracted internal stress state within RBC‐rich clots that compacted the RBCs, making the clot less porous and dictating the effectiveness of thrombolytics. Ho‐Tin‐Noe et al. [26, 27] expanded on these contractile clot properties, demonstrating the pre‐stressed state fibrin and RBCs undergo in contracted clots before lodging in large cerebral vessels. Kannojiya et al. [28] and Liu et al. [29] demonstrated the adhesive properties of clots in non‐physiologic environments, which may suggest a level of cohesion between a clot and an artery that ceases to experience physiologic flow. These cases illustrate the myriad factors that influence the outcomes of therapeutic techniques and are all considerations that need to be accounted for in computational modeling of AIS procedures.

The myriad unknowns in the process of vessel occlusion limit our understanding and applicability of benchtop experiments in improving patient outcomes. Computational fluid dynamics (CFD) studies have been used to characterize the embolization and transport of blood clots in aspiration thrombectomy studies [30, 31], yet these methods do not account for the compressive strain‐stiffening. Other groups have modelled the clot as a solid mesh of fibrin, which captures the extensional strain regimes but not compressive behaviors [32, 33, 34]. This is useful in knowing the risk of distal embolization, where clots may elongate and fracture in certain regions. Tutweiler et al. [35, 36], Rausch et al. [37], and Good et al. [38] demonstrated this dissipative property of solid‐like blood clots through internal and boundary level damage accumulation models. Good et al. [16], Oyekole et al. [39] and Patki et al. [40] modelled a viscoelastic clot with a fluid‐like, clot‐artery interface that failed at certain elongations in increasingly realistic arterial constitutive domains. While these models captured the behavior of the clot bulk properties, including the detachment tendencies during aspiration, they did not account for varied initial physiological conditions, including variations in the distal and proximal pressure differential (due to collateral circulation extent), varied vessel properties, internal stresses inherent in the arterial wall, and the resultant equilibrium state the clot experiences during vessel occlusion. These become initial conditions and therefore can drastically influence the outcomes of EVT simulations, meriting further investigation.

Given the complexity of the problem, understanding the arterially imposed stress state of a lodged clot in a physiological environment can provide insight into the internal forces that a clot actually experiences during mechanical thrombectomy. This challenge involves physics used for contact problems and cohesive interface problems. For example, an aged or calcified clot will most likely be more resistant to deformation and therapeutics than an uncontracted, unmineralized clot. The contraction of blood clots has been shown to increase the resistance to fracture and stabilize against distal embolization (c.f., e.g., [41, 42]). Characterizing the residual stress state in a clinical setting is very complex, but understanding the effects that these states can have on the outcomes of thrombectomy simulations is critical in realistic EVT calculations. In this study, we approach this problem from a numerical perspective. This study aims at investigating the influence of the initial conditions on the clot–arterial interaction, and how this influences the aspiration pressure at which the clot detaches from the vessel. We hypothesize that the residual stress state inherent in arteries, along with confounding factors such as collateral circulation, clot stiffness, and clot stress‐free size, will impact the reference configuration a lodged clot will experience in the cerebral vasculature. For this reason, this study outlines a formulation for a clot and artery that incorporates a hyperelastic clot embedded in a hyperelastic, fiber‐embedded artery.

## 2 Numerical Framework

### 2.1 Relevant Anatomy

**FIGURE 1:**
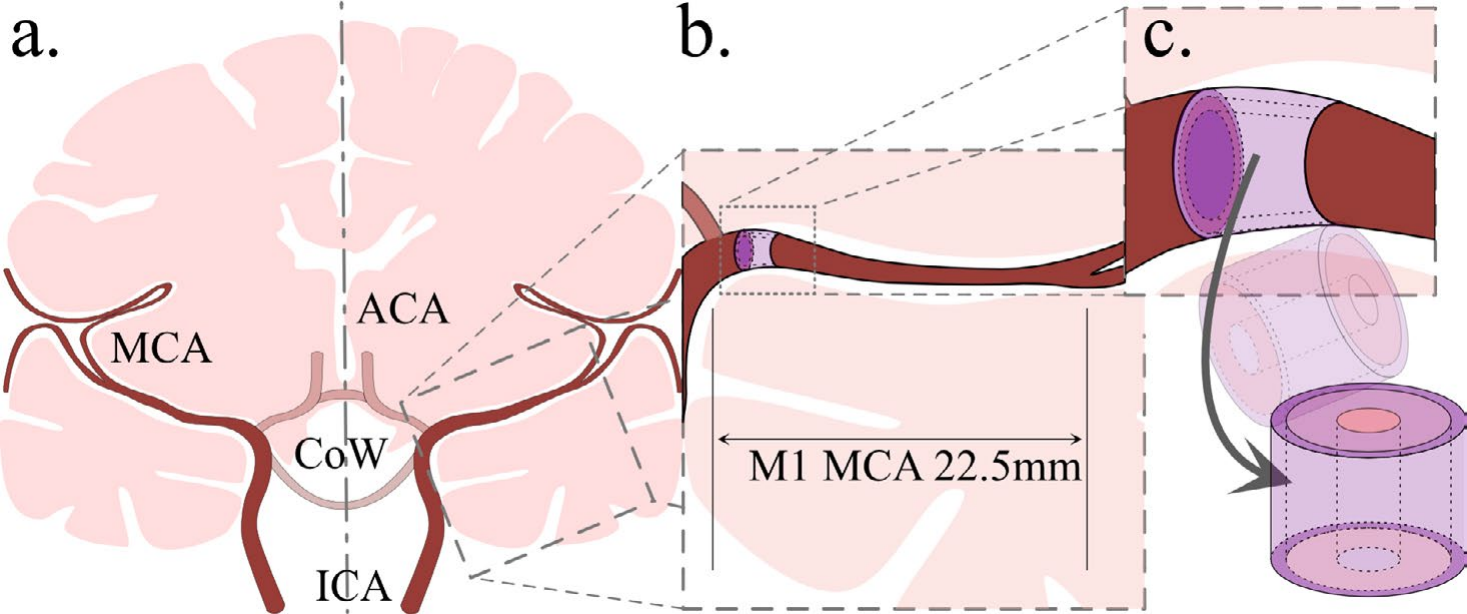
(a) Diagram of the central cerebral circulation with internal carotid arteries (ICAs), anterior cerebral arteries (ACAs), middle cerebral arteries (MCAs), and Circle of Willis. (CoW) (b) Zoomed in view of the M1 segment of the middle cerebral arteries, and (c) the representative two‐layered arterial section used in the computational domain, located at the proximal end of the M1 MCA. The M1 MCA is rotated 90 degrees in the computational domain, as depicted in the graphic. Figures are not to scale, exaggerated for illustration purposes.

Ischemic strokes predominantly occur in the internal carotid or M1 segment of the middle cerebral arteries (MCAs). The internal carotid arteries (ICAs) branch from the common carotid arteries (CCAs), connect to the Circle of Willis (CoW), and then into the M1 segment of the MCAs, as shown in Figure 1a. We will use the convention that regions closest to the CoW are proximal/upstream, and regions further from the CoW are distal/downstream. In this way, pressure differentials across a lodged embolus result from the difference between the proximal and distal mean arterial pressures on either surface of the clot. Following results from Rai et al. who measured MCA inner radii using ultrasound imaging [43], the M1 segment of the middle cerebral arteries is roughly 22.5 ± 8.1 mm long, with an inner radius of 3.1 ± 0.4 at the proximal end, as shown in Figure 1b. An axial pre‐stretch of 1% was applied to the artery to simulate in vivo axial stretches on most arteries [44, 45]. Due to the sensitive relative anatomy, wall thicknesses for the middle cerebral arteries are largely unknown. For this reason, material properties for internal carotid arteries of a rabbit for the Holzapfel‐Gasser‐Ogden model were scaled down, based on the ratio of inner diameters of internal carotid to middle cerebral arteries [46]. Wall thicknesses were assumed to be constant along the length of the MCA and were interpolated from measurements of intima‐media thicknesses and overall wall thicknesses of human common carotid arteries [47, 48, 49, 50, 51]. Base dimensions of the media, adventitia, and clot are listed in Table 1.

**TABLE 1:** Full parameter set for the closed artery geometry and material properties of the media, adventitia, clot, and aspiration pressure boundary conditions.

	Parameter	Value	Unit	Description and source
Media	$\rho_{m}$	1000	kgm3	Mass density of media, from Patki et al. [40]
	$\mu_{m}^{e}$	0.3	$\left[\mathrm{MPa}\right]$	Shear modulus of the media from Kroon and Holzapfel [52], Eriksson et al. [46], and Patki et al. [40]
	$k_{1,m}$	2.3632	$\left[\mathrm{kPa}\right]$	Fiber k1 parameter from Eriksson et al. [46] and Patki et al. [40]
	$k_{2,m}$	0.8393	—	Fiber k2 parameter from Eriksson et al. [46] and Patki et al. [40]
	$\beta_{f,m}$	29	$\left[{}^{\circ}\right]$	Orientation of collagen fibers in the media, from rabbit property, Eriksson et al. [46]
	$r_{m}$	1.55	$\left[\mathrm{mm}\right]$	Inner radius of the media, M1 MCA proximal end, from Rai et al. [43] and Mirza et al. [53]
	$l_{m}$	7	$\left[\mathrm{mm}\right]$	Reference configuration length of the media, M1 MCA, from Rai et al. [43]
	$t_{w,m}$	0.39	$\left[\mathrm{mm}\right]$	Wall thickness of the M1MCA, scaled down from CCA, Ciccone et al. [49] and Fernandes‐Alvarez et al. [48]
Adventitia	$\rho_{a}$	1000	kgm3	Mass density of adventitia, from Patki et al. [40]
	$\mu_{a}^{e}$	30	$\left[\mathrm{MPa}\right]$	Estimate from Kroon and Holzapfel [52], repeated in Eriksson et al. [46]
	$k_{1,a}$	0.5620	$\left[\mathrm{kPa}\right]$	Fiber k1 parameter from Eriksson et al. [46] and Patki et al. [40]
	$k_{2,a}$	0.7112	—	Fiber k2 parameter from Eriksson et al. [46] and Patki et al. [40]
	$\beta_{f,a}$	62	$\left[{}^{\circ}\right]$	Orientation of collagen fibers in the adventitia, Eriksson et al. [46]
	$r_{a}$	1.55	$\left[\mathrm{mm}\right]$	Inner radius of the adventitia, M1 MCA proximal end, calculated from media wall thickness
	$l_{a}$	7	$\left[\mathrm{mm}\right]$	Reference configuration length of the adventitia, M1 MCA, from Rai et al. [43]
	$t_{w,a}$	0.13	$\left[\mathrm{mm}\right]$	Wall thickness of the M1MCA, scaled down from CCA, Ciccone et al. [49] and Fernandes‐Alvarez et al. [48]
Clot	$\rho_{c}$	1080	kgm3	Mass density of the clot, from Patki et al. [40]
	$\eta_{c}$	8.33	$\left[\mathrm{Pa}*s\right]$	Dynamic shear viscosity of clot, from Patki et al. [40]
	$\mu_{G,c}^{e}$	4.36	$\left[\mathrm{kPa}\right]$	Gent elastic modulus for the clot, from Monclova et al. [14]
	$J_{\mathrm{mG},c}$	6.97	—	Gent chain stiffening parameter for the clot, from Monclova et al. [14]
	$r_{\text{cath}}$	0.9144	$\left[\mathrm{mm}\right]$	Inner radius of the aspiration catheter, for Navien 072 catheter, from Good et al. [16]
	$r_{c}$	4	$\left[\mathrm{mm}\right]$	Nominal outer radius of the clot
	$l_{c}$	4	$\left[\mathrm{mm}\right]$	Reference configuration length of the clot
Pressure BCs	$p_{\mathrm{px}}$	100	$\left[\text{mmHg}\right]$	Blood pressure in the artery and clot, proximal side, equal to MAP in MCAM1
	$p_{\mathrm{ds}}$	100	$\left[\text{mmHg}\right]$	Blood pressure in the artery and clot, distal side, is equal to MAP in MCAM1

For all resulting simulations, it was assumed that the clot lodged such that the proximal end is aligned with the origin of the M1 MCA, as shown in Figure 1c.

#### 2.1.1 Approach Overview

The basic premise of this study is that a clinician confronting AIS is presented with a clot lodged in an artery. The lodged clot and the arterial segment that contains it are in a state of equilibrium. In this state, neither the clot nor the artery is stress‐free. If the clot were excised from the artery, it would relax into a geometry different from that in the lodged state. The artery, whether with or without a lodged clot, is also characterized by a stress state of its own. The determination of the pre‐thrombectomy clot/artery state is a very complex problem. With this study, we hope to make a first contribution to the characterization of said state.

Our approach to the problem is based on basic concepts that have been used in the characterization of the residual stress state of arteries. Specifically, we assume that the artery and the clot each have their respective stress‐free states. Here

1. We consider a gallery of these stress‐free states.
2. We then posit a deformation process that places the clot within an arterial segment such that the clot lateral surfaces are in contact with the arterial inner wall.
3. We then study the achievement of a corresponding equilibrium state of the system.
4. Finally, we subject this equilibrium state to external pressure conditions that include the action of an aspiration catheter.

The geometry of the system's elements in their stress‐free states, along with their mechanical properties, is based on information available from the literature as well as measurements made in prior work by our group. The geometry into which we initially place the system, along with its corresponding mechanical state, is not, in general, an equilibrium state. The most severe assumption that we make is that once the clot and artery are placed together in the initial state, *they will deform to achieve an equilibrium state without sliding relative to one another*. Granted this assumption, the equilibrium state at point (iii) gives us an opportunity to assess what effect the system's residual stress might have on the ability to retrieve the clot by aspiration.

While we recognize the limitations of our study, we believe that this study is the first contribution of its kind and offers a framework for future refinements in the characterization of the stress state of a clot/artery system in the context of AIS.

### 2.2 Kinematics

**FIGURE 2:**
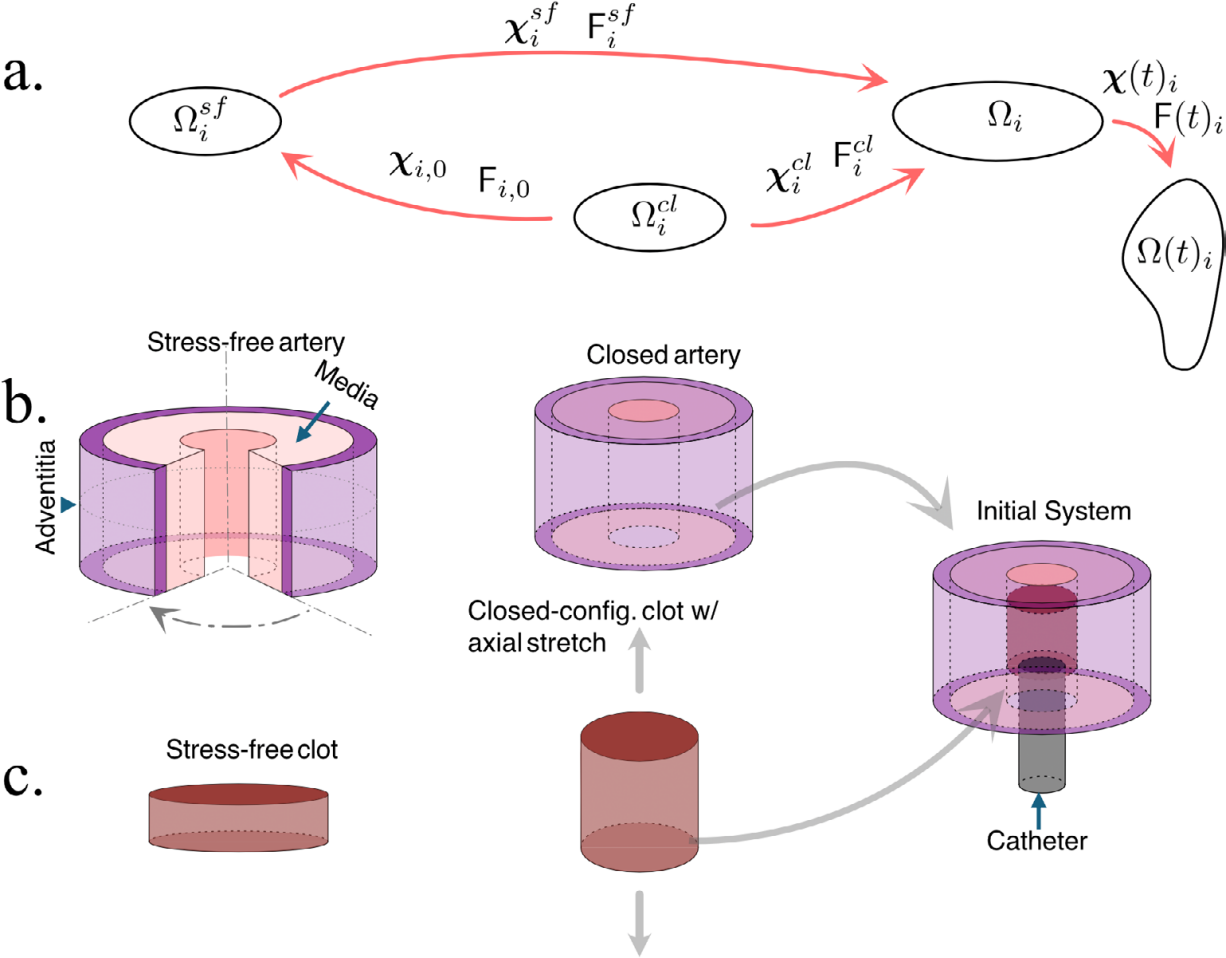
(a) Commutative diagrams showing the different configurations in the additive deformation sequence with (b) a representative image of an artery with an arbitrary opening angle, α, and the corresponding closed artery, and (c) the corresponding load‐free clot, its pre‐stretch, and insertion into the closed artery. Figures are not to scale, exaggerated for illustration purposes.

With reference to Figure 1c, we consider a segment of artery, and we assume that this segment is characterized by a residual stress state. We characterize the residual stress state by the opening angle theory of residual stress [54, 55, 56, 57, 58]. This is the idea that arteries have a stress‐free reference state that is revealed by (i) cutting the arterial wall with surfaces parallel to the artery's axis, and (ii) letting the artery relax thereby taking on the shape of an open, right‐cylinder with a specific opening angle [54, 55, 56, 57, 58]. The open shape is assumed to be stress‐free, and the residual stress of the closed artery can be estimated from a measure of the opening angle. To this framework, we add a concentric clot that is then lodged in the artery. The stress in the clot while in the artery can be estimated by the deformation that takes it from an assumed stress‐free state to the lodged state. In prior work, we modeled the contact between the clot and the arterial wall via a cohesive theory of interfaces [28, 39, 40, 59]. In this paper, we focus on the analysis of clot/artery equilibrium states under the assumption that the clot‐artery interface is fully coherent. Hence, a cohesive interface is not implemented here. For the same reason, we model the clot as hyperelastic, as opposed to viscoelastic as we had done in prior work. For each component of the system, there are four configurations: the initial or stress‐free/load‐free configuration ($\Omega_{i}^{\mathrm{sf}}$), the closed configuration ($\Omega_{i}^{\mathrm{cl}}$), the reference configuration ($\Omega_{i}$), and the current/deformed configuration $\Omega_{i}\left(t\right)$, the latter resulting from the applied aspiration (*i* = clot, media, adventitia), as shown in Figure 2. Following the convention of Tagiltsev et al., we then label particles in stress‐free (initial), closed, reference, and deformed configurations as $X_{i}^{\mathrm{sf}}$, $X_{i}^{\mathrm{cl}}$, $X_{i}$, and $x_{i}$, where ‘sf’ and ‘cl’, are the stress‐free and closed configurations for the *i*‐th component of the system (*i* = clot, media, adventitia) [60]. The gradient and divergence operators in all configurations are $\nabla_{j}$, and $\nabla_{j}\cdot$, respectively, with $j=X_{i}^{\mathrm{sf}}$, $X_{i}^{\mathrm{cl}}$, $X_{i}$, and $x_{i}$. The mapping of points between configurations is given as $X_{i}^{\mathrm{sf}}=\chi_{i,0}\left(X_{i}^{\mathrm{cl}}\right),\;X_{i}=\chi_{i}^{\mathrm{sf}}\left(X_{i}^{\mathrm{sf}}\right)$, $x_{i}=\chi_{i}\left(X_{i}\right)$ for the stress‐free, reference, and current configurations, respectively. Likewise, the deformation gradients ($F_{i}^{j}$ = $\nabla x_{j}$, *j* = $X_{i}^{\mathrm{sf}}$, $X_{i}^{\mathrm{cl}}$, $X_{i}$, $x_{i}$) are given by $F_{i}$, $F_{i,0}$, and $F_{i}^{\mathrm{sf}}={F_{i}F_{i}^{\mathrm{cl}}F}_{i,0}^{-1}$ for the reference, closed, and stress‐free configurations, respectively. The general displacement and the Jacobian of the deformation gradient, and the left and right Cauchy‐Green strain tensors are given by $w_{i}=x_{i}-X_{i}$, $J_{i}=\det\left(F_{i}\right)$, $B=FF^{T}$, and $C=F^{T}F$. We note that Figure 2 shows idealized geometries, of the system, where the clot and the artery are presented as right cylinders, when in reality, in the initial configuration, the clot and artery are allowed to equilibrate and would not have such regular geometries.

### 2.3 Constitutive Models (Clot, Artery, Interface)

#### 2.3.1 Artery

The selected artery segment (Figure 1c) was modeled as a two‐layer right cylinder. The artery was assumed to be an incompressible, hyperelastic solid, following work by Gasser et al. [61, 62] and Holzapfel et al. [63], shown in Equation (1), and the Helmholtz free energy (${\tilde{\psi }}_{i}$) given by Equation (2):

(1) $$P_{i}=p_{i}F^{-T}+2F\frac{\partial {\tilde{\psi }}_{i}}{\partial C}$$

(2) $${\tilde{\psi }}_{i}=\frac{\mu_{i}}{2}\left(I_{1}\left(\bar{C}\right)-3\right)+\frac{k_{1,i}}{2k_{2,i}}\sum_{j=4,6}\left(e^{k_{2,i}{\left(I_{j}\left(\bar{C}\right)-1\right)}^{2}}-1\right)$$

where $P_{i}$ is the first Piola‐Kirchhoff stress tensor for the *i*‐th component of the artery, $p_{i}$ is the multiplier for the enforcement of the incompressibility constraint, and ${\tilde{\psi }}_{i}$ is the Helmholtz free energy for the *i* = media, adventitia. C is the right Cauchy‐Green strain tensor, $\bar{C}={\left(\det\left(C\right)\right)}^{-1/3}C$ is the isochoric component of C. In the free energy expression, $\mu_{i}$ is the shear modulus (similarly to what is found in a neo‐Hookean model), $k_{m,i}$, (*m* = 1,2, *i* = media, adventitia) are fiber stiffness parameters, and $I_{j}\left(\bar{C}\right)$ (*j* = 4,6) are the pseudo‐invariants of $\bar{C}$ that account for the direction of the fibers embedded in the elastic matrix. Specifically, $I_{4}\left(\bar{C}\right)=m_{1}\cdot \bar{C}m_{1}$ and $I_{6}\left(\bar{C}\right)=m_{2}\cdot \bar{C}m_{2}$, with $m_{1}$ and $m_{2}$ being the unit vector fields that describe the fiber orientation in the initial configuration of the first and second fiber family, respectively. In this arterial representation, we assume the artery to have two layers, with the tunica intima having negligible thickness relative to that of the other arterial layers [55, 64].

Following work by Holzapfel et al. [61] and Tagiltsev et al. [60], among others (c.f., e.g., [65, 66, 67, 68]), the closed state (cf. Figure 2b–center) has the geometry of a closed right cylinder. This state is assumed to have been achieved by deforming an open geometry (Figure 2c–left) that is also assumed to be a right cylinder. In Tagiltsev et al. [60], using a cylindrical coordinate system with a vertical axis coinciding with the axis of the artery, the deformation in question can be expressed as follows:

(3) $$\zeta_{\mathrm{sf}}=k\sqrt{\frac{\left(R^{2}-R_{i}^{2}\right)/\mathrm{kc}+r_{i}^{2}}{R}}$$

(4) $$\left[F_{i}^{\mathrm{sf}}\right]=\left[\begin{array}{ccc} c\zeta_{\mathrm{sf}} & 0 & 0\\ 0 & \frac{1}{\zeta_{\mathrm{sf}}} & 0\\ 0 & 0 & \frac{1}{c} \end{array}\right]$$

where R and r denote the radial coordinate in the open and in the closed states, respectively, the subscript i stands for “inner”, $\zeta_{\mathrm{sf}}$ is the mapping of points from the stress‐free (*sf*) state of the system to the closed (*cl*) state, $k=2\pi /\left(2\pi -\alpha \right)$ is the parameter associated with the opening angle, α of the cut artery, and c=l/L is the nondimensional ratio of lengths between the two states of the system (see Equations (24) and (25) from Tagiltsev et al. [60]). The fiber orientation relative to the z‐axis (in a cylindrical coordinate system), the inner, intermediate, and outer radii, and the height of the specimen are all defined in Table 1.

#### 2.3.2 Clot Stress State

In Patki et al. and Monclova et al., we modeled the clot as viscoelastic with features similar to those of a Kelvin‐Voigt solid [14, 40]. Specifically, the clot's second Piola‐Kirchhoff stress tensor (S), and associated elastic strain energy were given by, Equations (5) and (6), respectively:

(5) $$S=-pC^{-1}+S^{e}+S^{v}$$

(6) $${\tilde{\psi }}_{G}=-J_{m}\frac{\;\mu_{c}}{2}\;\ln\left(\frac{I_{1}\left(\bar{C}\right)-3}{J_{m}}\right)$$

where $S^{e}$ is the elastic stress contribution derived from a Gent hyperelastic model (${\tilde{\psi }}_{G}$), and $S^{v}$ is a viscous contribution to the overall stress. For the free energy of Gent's model, $J_{m}$ is the parameter associated with chain limited stiffening, and $\mu_{c}$ is the shear modulus of the clot. In this study, we consider only equilibrium states of the clot. As such, the viscous component of the clot's stress response is equal to zero. What is most important in this study is the fact that the clot is assumed to have an initial stress‐free configuration whereas the lodged clot is characterized by a externally imposed stress state. The stress‐free state is depicted in Figure 2c–left. The clot is then deformed into the geometry shown in Figure 2c–center such that the clot can be placed within, and in contact, with the closed artery. The deformation taking the system from the state in Figure 2c–left to that in Figure 2c–center has the following deformation gradient in Equation (7):

(7) $$\left[F_{c,0}\right]=\left[\begin{array}{ccc} \frac{1}{\sqrt{\eta_{\mathrm{sf}2\mathrm{cl}}}} & 0 & 0\\ 0 & \frac{1}{\sqrt{\eta_{\mathrm{sf}2\mathrm{cl}}}} & 0\\ 0 & 0 & \eta_{\mathrm{sf}2\mathrm{cl}} \end{array}\right]$$

which corresponds to a uniaxial, volume‐preserving stretch in the axial direction. The deformation gradient in Equation (7) is completely described by the ratio of the clot length in the stress‐free state to that of the clot in the initial state. In order for the clot to be lodged, we assume that the clot radius is initially greater than or equal to the effective reference vessel inner radius. As already mentioned, while blood clots are generally considered to be viscoelastic, the calculations were done to determine equilibrium states of a clot, as would appear in AIS patients, pre‐surgery, and therefore, all rate‐dependence in the calculations is neglected.

### 2.4 Incompressibility Constraint and Momentum Balance Equations

**FIGURE 3:**
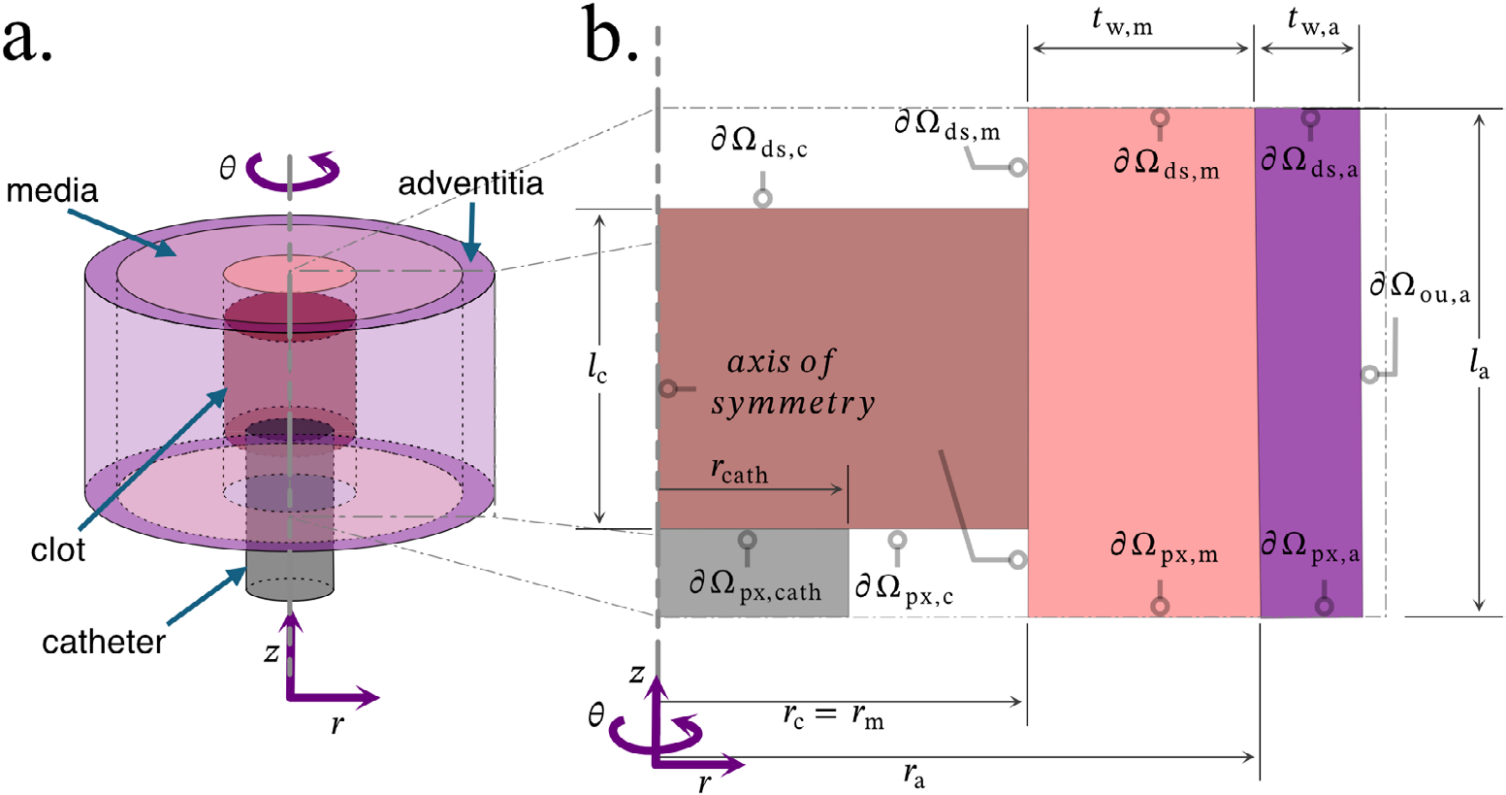
(a) 3D view of the reference geometry, $\Omega_{i}^{\mathrm{cl}}$, of the closed artery with a concentric clot and aspiration catheter. (b) The axisymmetric 2D view of closed artery with the corresponding clot located concentrically inside, and an aspiration catheter applying a suction at the proximal (lower) end of the artery. $t_{w,i}$ stands for the wall thickness, $r_{i}$ are the radii, $l_{i}$ are the lengths, and ${\partial \Omega }_{j,i}$ are the boundaries for the *j*‐th regions of the *i*‐th components of the system (*j* = ds‐distal, px‐proximal, di‐distal inner, pi‐proximal inner, ou‐outer, *i* = cath‐catheter, c‐clot, m‐media, a‐adventitia). The axis of symmetry is shown on the left of the 2D diagram. Diagram not to scale.

The resultant initial/closed geometry ($\Omega_{i}^{\mathrm{cl}}$) is then shown in Figure 3b. The equilibrium equation for the clot, media, and adventitia from the initial configuration $\Omega_{i}$ and the incompressibility constraint are given by Equations (8) and (9):

(8) $$0=\nabla_{X_{i}}\cdot P_{i}$$

(9) $$J_{i}-1=0$$

where *i* = clot, media, adventitia. The weak form implementations of these equations are used to solve for each equilibrium state of the system, neglecting inertial terms. To handle large boundary loads, a numerical continuation method (cf. [69]) was used with different continuation parameters depending on the calculation, for example the opening angle.

### 2.5 Boundary and Initial Conditions

Figure 3 depicts the 3D artery and subsequent 2D axisymmetric view of the closed geometry with naming conventions used and boundary conditions listed. The outer arterial wall was assumed to be traction‐free, as shown in Equation (10):

(10) $$\sigma \cdot n=0\;\mathrm{on}\;{\partial \Omega }_{\mathrm{ou},a}$$

where ‘*ou,a*’ designates the outer, adventitia component of the system. Mean arterial pressure was prescribed to the proximal clot (‘*px,c*’), distal clot (‘*ds,c*’), proximal inner media (‘*pi,m*’), and distal inner media (‘*di,m*’) boundaries, as shown in Figure 3b, and given by Equations (11) and (12):

(11) $$\sigma \cdot n=p_{\mathrm{px}}n\;\mathrm{on}\;{\partial \Omega }_{\mathrm{pi},m}\;\text{and}\;{\partial \Omega }_{\mathrm{px},c}$$

(12) $$\sigma \cdot n=p_{\mathrm{ds}}n\;\mathrm{on}\;{\partial \Omega }_{\mathrm{di},m}\;\text{and}\;{\partial \Omega }_{\mathrm{ds},c}$$

Base proximal and distal pressure values ($p_{\mathrm{px}}$ and $p_{\mathrm{ds}}$) of 100 mmHg mean arterial pressure were prescribed according to previous work [40]. We assume that the clot is not in full contact with the aspiration catheter, and so we model the applied aspiration on a portion of the proximal clot boundary, ${\partial \Omega }_{\mathrm{px},\text{cath}}$, as shown in Equation (13):

(13) $$\sigma \cdot n=-p_{\mathrm{asp}}n\;\mathrm{on}\;{\partial \Omega }_{\mathrm{px},\text{cath}}$$

For a static aspiration of magnitude $p_{\mathrm{asp}}$ given in Table 2. Roller conditions on the proximal and distal arterial walls were set to restrict the axial movement of the artery, as given by Equation (14):

(14) $$w\cdot n_{z}=0\;\mathrm{on}\;{\partial \Omega }_{\mathrm{px},m},{\partial \Omega }_{\mathrm{px},a},{\partial \Omega }_{\mathrm{ds},m},\text{and}\;{\partial \Omega }_{\mathrm{ds},a}$$

for the proximal media and adventitia (‘px,m’, ‘px,a’) and distal media and adventitia (‘ds,m’, ‘ds,a’), respectively. Here, w is the displacement, and $n_{z}$ is the axial outward unit normal. In this way, the boundary system allowed for radial expansion or contraction of the artery dependent on the reference state of the clot. Because the clot is considerably more deformable than the artery, when the artery/clot systems achieve their equilibrium states, it is not uncommon for the proximal and distal surfaces of the clot to bulge so to contact with the arterial wall. The possibility of said contact was accounted for using the contact solution scheme available in COMSOL Multiphysics [70] based on an augmented Lagrangian method [71]. Finally, the initial displacement and multiplier conditions were given as in Equation (15):

(15) $$w_{i}\left(X_{i}\right)=0,\text{and}\;p_{i}\left(X_{i}\right)=0\forall X_{i}\in \Omega_{i}$$

for the i‐th component of the system (*i* = clot, media, adventitia). We note that the problem we consider is static. We note that in this instance, the subscript ‘i’ denotes the initial configuration of the system, using the labeling system above, rather than an indication of an initiation of a time stepping scheme. That is, the i stands for the initialization of the nonlinear (static) solver.

**TABLE 2:** Full parameter set for parameter sweeps performed in the simulation dataset.

Parameter	Base value	Range	Units	Description
pdsppx	1	1–0.1	—	Clot distal to proximal pressure ratio
lsflcl	0.6	0.2–1.6	—	Stress‐free to closed length of the clot
$l_{\mathrm{cl},\text{clot}}$	4	2–24	$\left[\mathrm{mm}\right]$	Computational length of the clot in the closed configuration
$\mu_{G,c}^{e}$	2 × 4.36	0.4–8.4	$\left[\mathrm{kPa}\right]$	Gent shear modulus for the clot
$\alpha_{\text{media}}$	165	0–200	$\left[{}^{\circ}\right]$	Opening angle of the media, set equal to the adventitia opening angle, similar to Tagiltsev et al. [60]
$\beta_{f,a}$	62	0–60	$\left[{}^{\circ}\right]$	Media/adventitia fiber orientation
$r_{i,\mathrm{sf}}$	3.0225	0.0225–3.0225	$\left[\mathrm{mm}\right]$	Artery stress‐free inner radius
$p_{\text{cath}}$	—	0–900	[mmHg]	Aspiration pressure applied to the clot

*Note:* Base values are values held constant before the parametric sweep; ranges were conducted at 5% increments of the maximum range of sweeps.

### 2.6 Finite Element Implementation

The simulations were run as static calculations, ignoring inertial and body force terms. Deformations were applied to the initial configuration using the ‘Predeformation’ node in COMSOL Multiphysics [70]. Said node provides a convenient user interface for the composition of the deformation gradient of the current configuration (i.e., that relative to the initial configuration) with the deformation gradient of the deformation taking the system from its stress‐free state to the initial state. The deformation gradient that appears in the strain energy, along with its associated stresses and moduli, is that produced by said composition. A direct stationary solver node was used to solve for internal deformation points with a MUMPS solver.

## 3 Results

**FIGURE 4:**
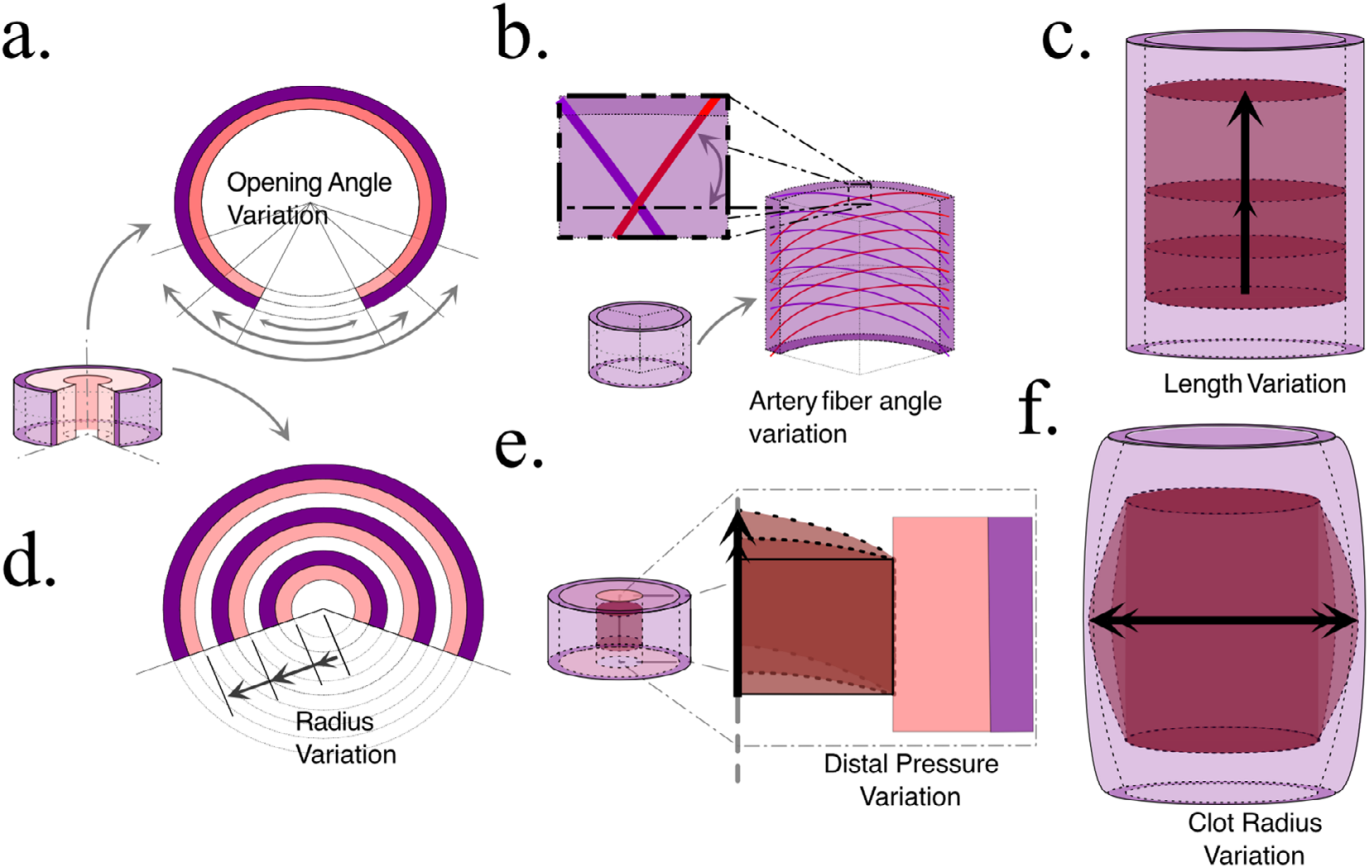
(a) Schematic showing successive variations in the opening angle of the media of the stress‐free artery, $\alpha_{\text{media}}$. (b) Successive variations in the fiber angle of the media of the stress‐free artery, $\beta_{f,m}.$ (c) Success lengthening of the clot in the initial configuration, $l_{\text{comp},\text{clot}}$, (d) successive variations in the inner radius of the media of the stress‐free artery, $r_{i,\mathrm{sf}}$. (e) Successive variations in distal pressure ($p_{\mathrm{ds}}$) relative to the proximal pressure ($p_{\mathrm{px}}$) on the clot in the reference state, and (f) successive widening of the clot as a result of variations in the stress‐free to closed‐configuration lengths of the clot, lsflcl. Figures are not to scale, exaggerated for illustration purposes.

Overall, parameter sweeps were performed holding all other parameters constant, to calculate the stress state of the reference artery, clot, and lodged clot, as shown in Table 2 and Figure 4.

For each of the initial parameter sweeps, a sweep of aspiration pressures ranging from 0 to 900 *mmHg* was completed to calculate the overall stresses in the system during aspiration thrombectomy. Figure 4 shows representative parameter sweeps of the material, such as the opening angle, $\alpha_{\text{media}}$ of the stress‐free media (set equal to the opening angle of the adventitia) (Figure 4a), the inner radius of the stress‐free media (Figure 4b), the fiber orientation of the media (Figure 4c), and the pressure ratio across the proximal‐distal sides of the clot (Figure 4d). Additional sweeps were performed for increasing reference length of the clot, stress‐free to reference length ratio of the clot, and clot shear modulus, $\mu_{G,c}^{e}$, with ranges shown in Table 2. Successive aspiration pressures were then applied to ${\partial \Omega }_{\mathrm{px},\text{cath}}$, the proximal portion of the clot boundary equal to the radius of a standard Navien 072 catheter.

### 3.1 Stress State of the Artery

**FIGURE 5:**
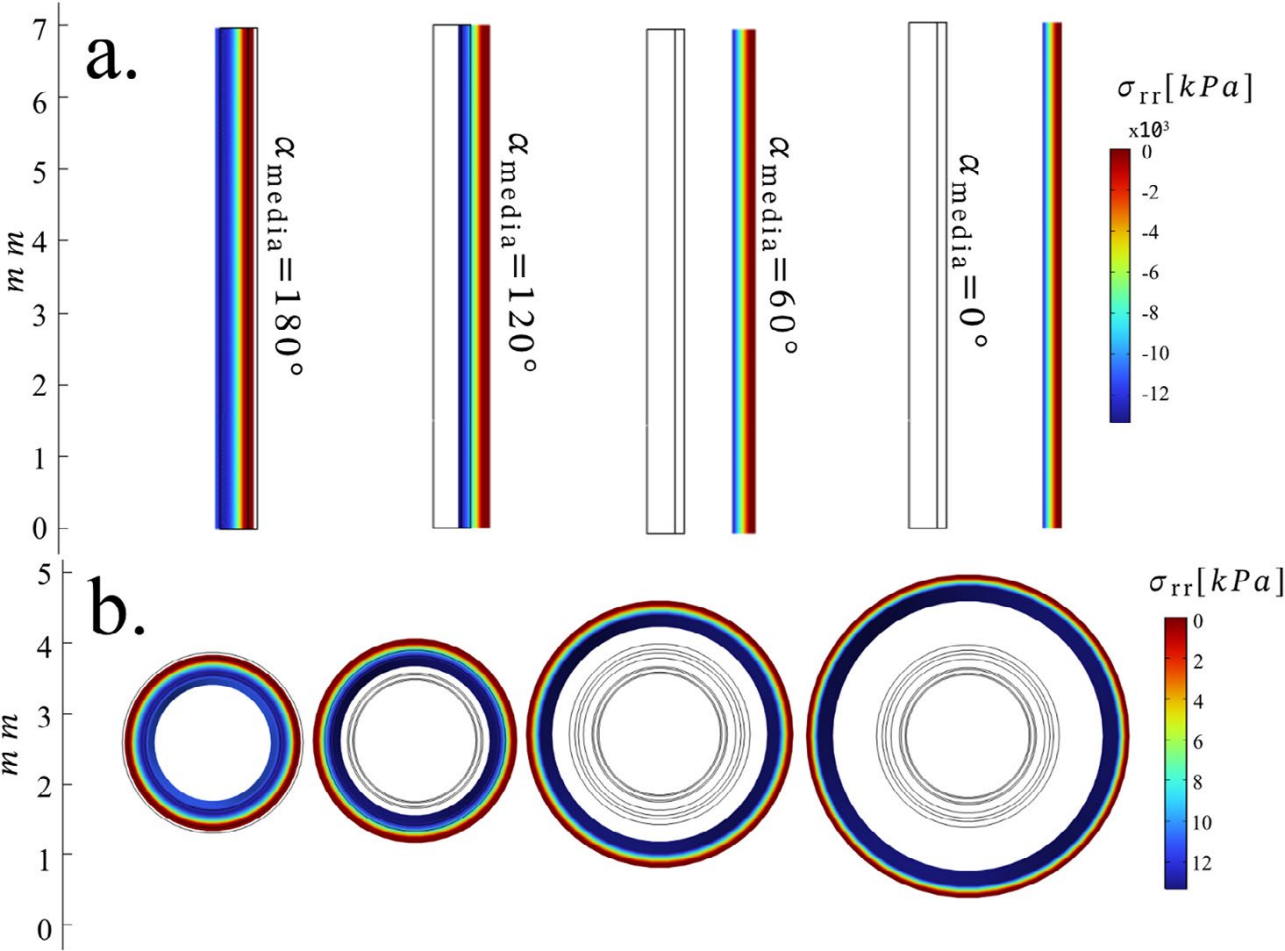
(a) Successive reference configurations of the media and adventitia wall, with stress‐free media opening angles $\alpha_{\text{media}}=0^{{}^{\circ}},60^{{}^{\circ}},120^{{}^{\circ}},\text{and}\;180^{{}^{\circ}}$, showing the rz‐plane in an axisymmetric framework, and (b) the corresponding r θ‐plane, with outline showing the ‘geometric’ configuration in COMSOL, what we call $\Omega_{i}^{\mathrm{cl}}$. Outlines depict the closed‐configuration boundaries. Here, the mean arterial pressure is applied on the internal artery surface −13.3 kPa (100 mmHg).

Figure 5 shows the results of axisymmetric calculations for the determination of the equilibrium state of the artery under the action of an internal pressure of −13.3 kPa (100 mmHg) and under an initial stretch (from stress‐free) of 1%. Views are presented in both the rz‐ and *r* θ‐ planes. It is known that the radial component of the stress in an artery is only weakly dependent on the opening angle [55, 72, 73, 74]. Here we report it because it plays an important role in how a clot can be detached from the artery. It is for this reason that we indicated the radial component of the stress in all stress plots, namely, to highlight the interplay between the radial detachment and the resulting traction distributions near the clot‐artery interface, the latter being the primary focus of this study. The plots display the radial component of the Cauchy stress and the position of the arterial wall against the initial position (wireframe, to scale) of the same, as functions of the artery's stress‐free opening angle. In this calculation, the media stress‐free opening angle $\alpha_{\text{media}}$, is decreased from $180^{{}^{\circ}}$ (base value) to $0^{{}^{\circ}}$, holding the radius and wall thickness of the closed artery fixed. When the artery's equilibrium state is achieved, the artery displaces outward as $\alpha_{\text{media}}$ is decreased from $180^{{}^{\circ}}$ (flat sheet) to $0^{{}^{\circ}}$ (fully closed in the initial state). The result shows an artery whose inner wall is in a compressive state (radially) that decreases in magnitude as the angle decreases. For reference, the equilibrium luminal radii at 100 mmHg were approximately 3.02, 3.96, 5.63, and 7.00 mm for $180^{{}^{\circ}}$, $120^{{}^{\circ}}$, $60^{{}^{\circ}}$, and $0^{{}^{\circ}}$ respectively. These values highlight how the increasing opening angle decreases the loaded lumen radius, consistent with the trends shown in Figure 5. The wall thickness noticeably decreases as the artery distends outward with respect to the closed configuration. In a similar calculation, the fiber orientation of the media and adventitia is decreased from their base values, measured by Eriksson et al. [46] to $0^{{}^{\circ}}$ fully perpendicular to the axial direction, with the artery contracting inward slightly with lower and lower fiber angles. A third calculation depicting the variation of the inner radii of the stress‐free artery with the initial configuration shows a proportional decrease in inner radius as the stress‐free inner radius is decreased.

### 3.2 Stress State of the Clot

**FIGURE 6:**
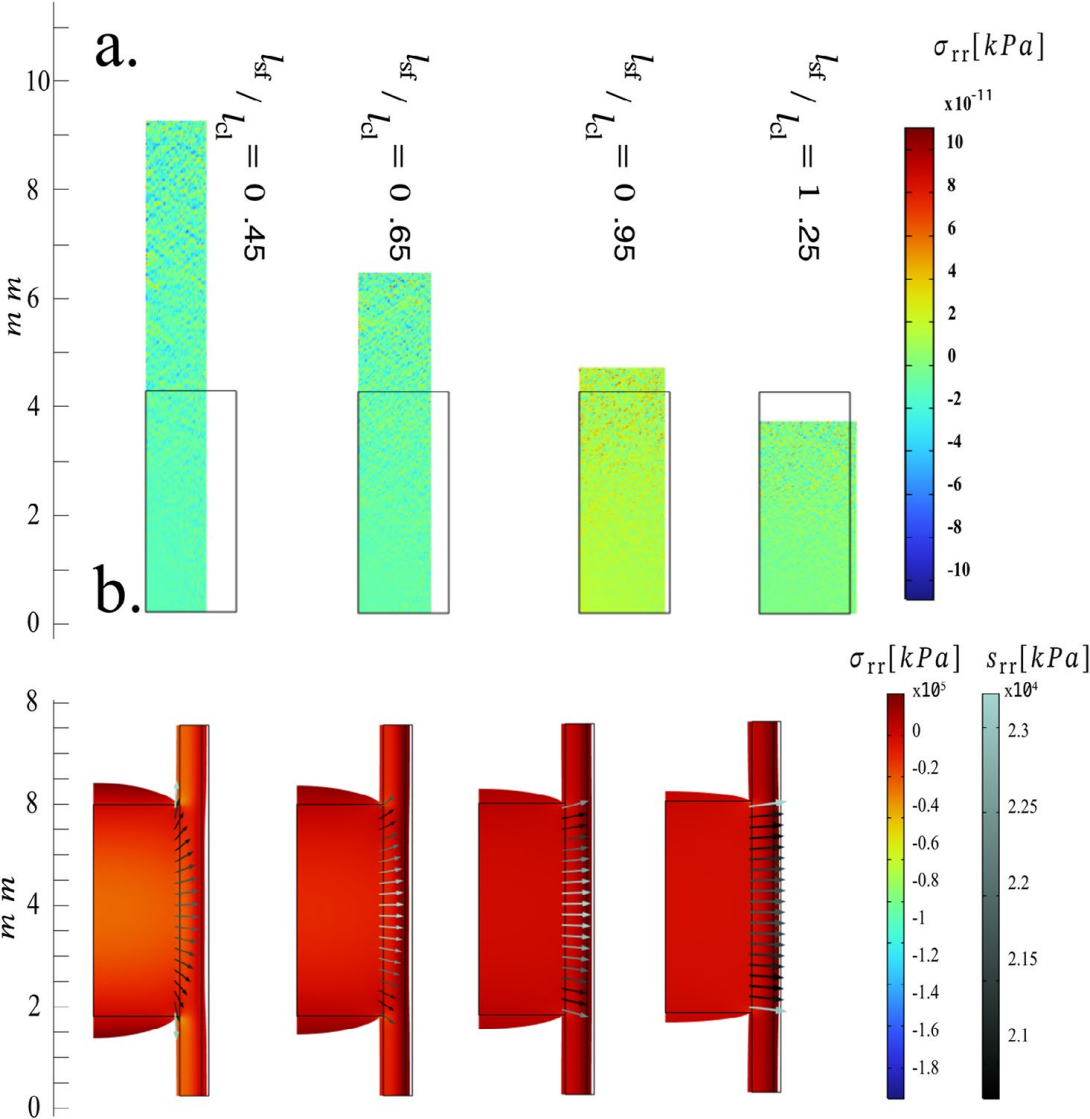
(a) Successive closed‐artery configurations of the clot, with stress‐free to closed length of the clot lsflcl=0.4,0.65,0.95,1.25, showing the rz‐plane in an axisymmetric framework, and (b) the corresponding clot lodged in the artery in the initial configuration, in COMSOL. $\sigma_{\mathrm{rr}}$ is the radial component of the Cauchy stress and $s_{\mathrm{rr}}$ is the traction of the clot onto the artery. Outlines depict the closed‐configuration boundaries.

Four variables were changed separately in the clot, as shown in Table 2, the length of the closed clot, $l_{\mathrm{cl},\text{clot}}$, the ratio of stress‐free to initial lengths.

lsflcl, the modulus of the clot, $\mu_{G,c}^{e}$, and the pressure difference across the clot, pdsppx (ratio of distal to proximal mean arterial pressures). Figure 6a shows a representative referential image of successive increases in stress‐free to closed‐configuration length of the clot, with the closed‐configuration clot geometry outlined in the image. As the ratio increases, the equilibrated clot becomes shorter and wider with respect to the initial configuration, representing the shape the clot would take if it were to be removed from the artery intact, as shown in Figure 6a. The same calculation was performed with the artery included in which the outline shows the closed configuration, the shaded region depicts the equilibrated region, and the arrows between the clot and artery depict a compressive traction from the clot into the arterial wall that increases in magnitude with increased stress‐free to closed‐configuration length ratio. As expected, the clot bulges axially due to its relative radius to the artery and presses firmer into the arterial wall as the ratio increases, as shown in Figure 6b. This demonstrates the change from the idealized geometry used in the stress‐free configuration, where the overall shapes are right‐cylindrical. Similarly, as the clot modulus is increased from that of a relatively stiff clot, such as those in works by Boodt et al. [15] to that of a collagenous plaque embolus, the compressive tractions increase into the arterial wall, demonstrating a higher stress state overall with a stiffer clot of the same size, as shown in the Figure S1.

**FIGURE 7:**
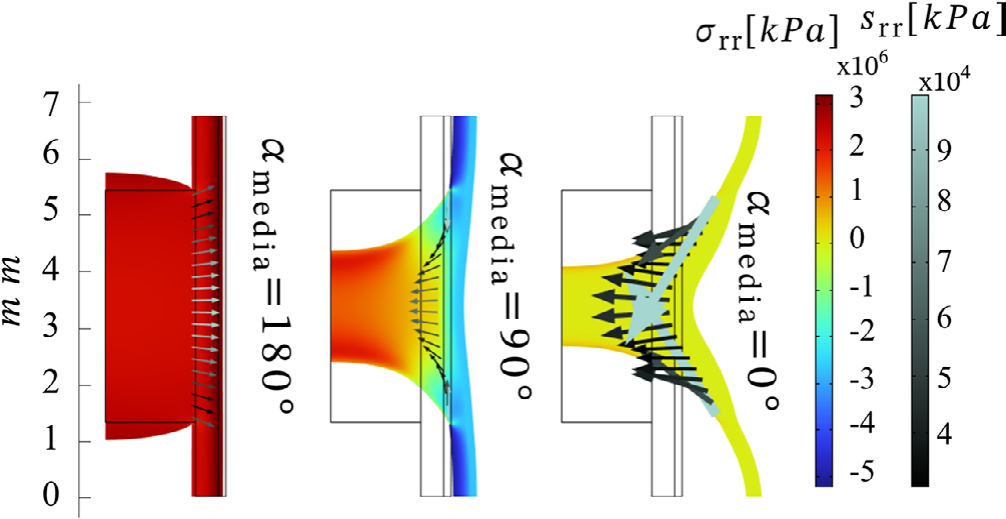
Successive reference configurations of the media and adventitia wall, with stress‐free media opening angles $\alpha_{\text{media}}=180^{{}^{\circ}},90^{{}^{\circ}},\text{and}\;0^{{}^{\circ}}$, showing the rz‐plane with the clot causing the arterial wall to distend inward with respect to the closed configuration, $\Omega_{i}^{\mathrm{cl}}$. $\sigma_{\mathrm{rr}}$ is the radial component of the Cauchy stress and $s_{\mathrm{rr}}$ is the traction of the clot onto the artery. Outlines depict the closed‐configuration boundaries.

In a separate set of calculations, the distal pressure on the clot and the opening angle of the stress‐free artery were varied, and the system allowed to equilibrate. As the distal pressure on the clot was decreased from mean arterial pressure, 100 to 0 mmHg, the clot displaced distally with respect to the closed configuration. This would represent a case of poorer and poorer collateral circulation, creating a pressure differential across the clot, causing it to distend distally in its initial configuration. This had the effect of also decreasing the magnitude of the traction of the clot into the arterial wall on the distal side of the clot, creating a subsequent, non‐symmetric gradient of tractions from proximal to distal ends of the clot. As the opening angle of the artery increased, the clot, which was assumed to be continuously connected with the interface, distended outward with the arterial wall, corresponding to the increase in closed‐configuration radius of the artery from Figure 5. For opening angles smaller than $165^{{}^{\circ}}$, the clot‐artery interface was in a purely tensile state, as shown in Figure 7. While not physically meaningful, this set of calculations show the consideration needed when choosing initial conditions for the stress state of the clot lodged in an artery. For opening angles that have been measured in carotid arteries (*c.f*., e.g., [46, 66, 75]), namely values greater than $165^{{}^{\circ}}$ for arteries smaller than the CCAs, the opening angle yields a residual stress state for the reference configuration similar in radius to the closed configuration and would therefore produce a compressive state for clots of the radii considered here (the size of clots extracted from AIS patients).

### 3.3 Aspiration EVT and Clot Detachment

**FIGURE 8:**
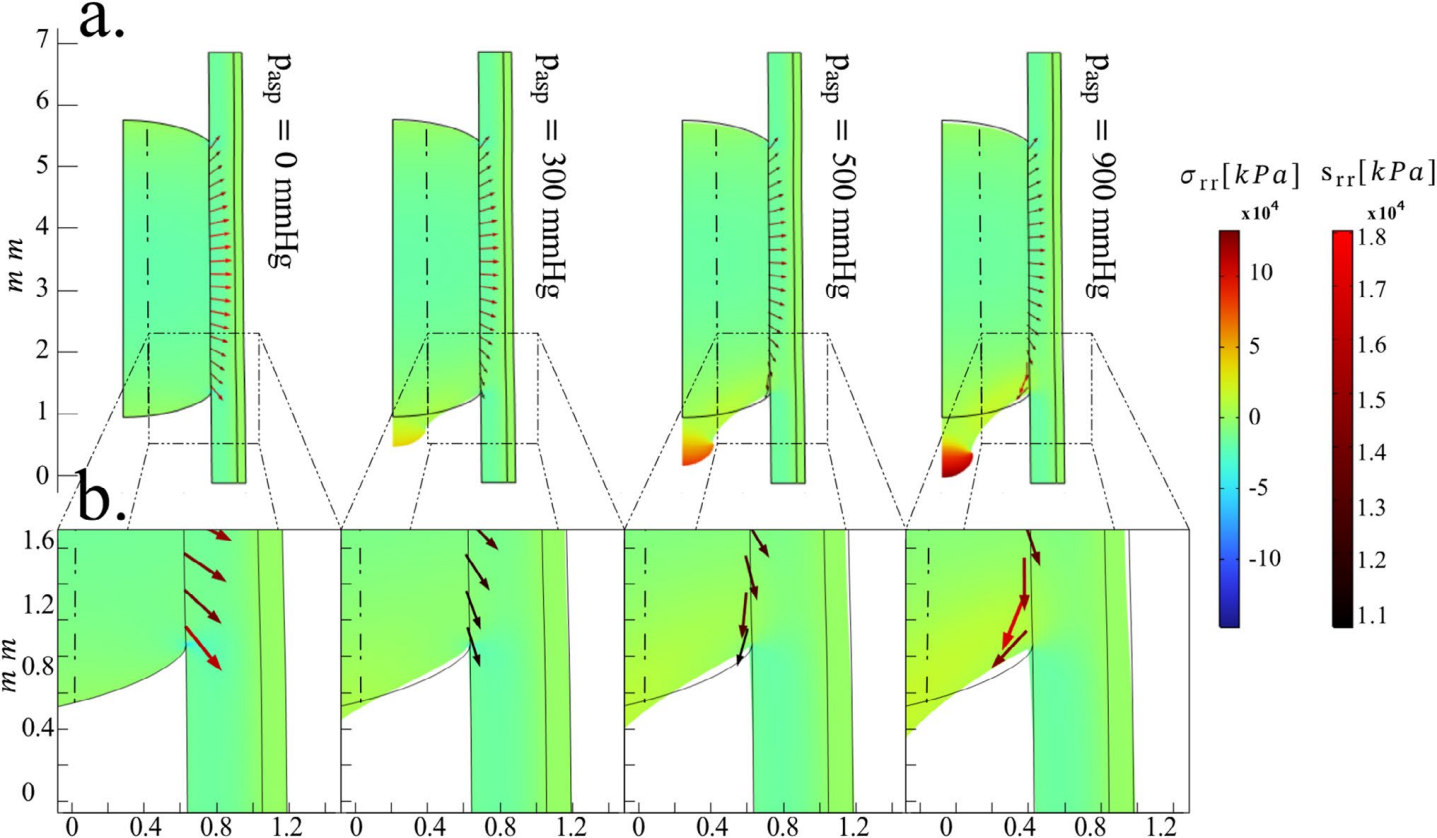
(a) Successive reference configurations of the artery and clot, with successive aspiration pressures applied on ${\partial \Omega }_{\mathrm{px},\text{cath}}$, with relative aspiration pressures of $p_{\mathrm{asp}}=0,300,500,\text{and}\;900\;\text{mmHg}$, showing the rz‐plane in an axisymmetric framework, with the outline corresponding to the reference configuration $\Omega_{i}$ and the aspiration applied in the deformed configuration. (b) The corresponding zoomed in view of the clot artery interface on the proximal end with increasing aspiration pressures, showing the traction vectors (arrows) turning from compressive (into the artery) to tensile (beginning to detach). $\sigma_{\mathrm{rr}}$ is the radial component of the Cauchy stress and $s_{\mathrm{rr}}$ is the traction of the clot onto the artery. Outlines depict the closed‐configuration boundaries. Dashed vertical line represents the radial location of the catheter inner diameter.

After considering the stress state of a lodged clot in a pre‐stressed artery, we considered the effect of these sweep of variables on the aspiration pressure necessary to begin removing the clot. As an example, Figure 8a shows the clot with base dimensions in its initial, equilibrium state, and an aspiration pressure is applied to the portion of the proximal clot boundary, ${\partial \Omega }_{\mathrm{px},\text{cath}}$. The aspiration pressures considered ranged from 0 to 900 mmHg, encompassing the range of aspirations used in aspiration EVT [30, 38, 39, 76]. Figure 8b shows the resultant zoomed in proximal clot‐artery interface, with successive increasing aspiration pressures, and the reference geometry outlined in black. As the aspiration pressure increased, the clot near the aspiration was proximally displaced, to the point where the traction vectors turn from compressive to tensile, indicating a proclivity of the clot to detach. This metric was used to determine the effect of each of the parameter sweeps discussed earlier, to interpolate the aspiration pressure necessary to begin removing a clot. The resultant tractions of the clot into the artery were calculated for each simulation sweep, and the point at which the tractions became zero were interpolated in MATLAB, using an inbuilt interpolation function.

**FIGURE 9:**
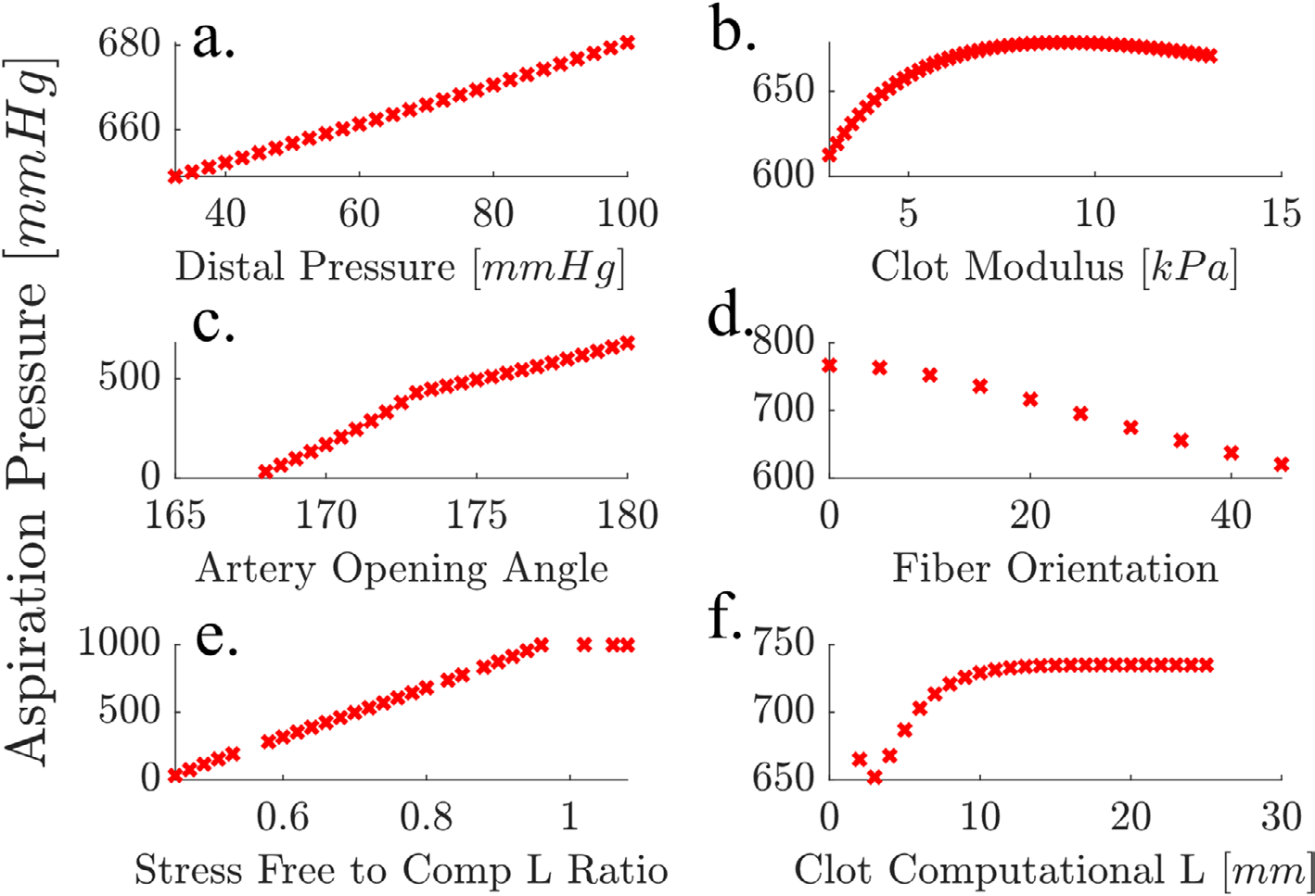
Aspiration pressures necessary to cause a tensile load on the clot‐artery interface given various parameter sweeps.

From Figure 9, we observe the resultant aspiration pressures required to begin detaching the clot from the artery, as different variables in the initial configuration are modified. Figure 9a shows a linear decrease in the removal pressure as the distal pressure on the clot decreases, with pressures of 340 and 320 mmHg needed to remove a clot who's distal to proximal pressure ratios decrease from 1 to 0. Similarly, as the clot modulus increases 13‐fold, the removal pressures increase from 605 mmHg to nearly 675 mmHg, and then nonlinearly decrease to around 650 mmHg. As the artery opening angle decreased, the clot in the initial configuration was loaded in a tensile state, as previously discussed. With a nominal opening angle of 180, the necessary removal pressure was roughly 620 mmHg. The fiber orientation in the media played a significant role in the resultant aspiration removal pressures, with pressures decreasing from nearly 600 to approximately 400 mmHg as the fiber orientation increased from 0° (parallel with the r‐axis) to 100° from the horizontal. This suggests that the artery plays a large role in the extraction pressures in AIS. As the stress‐free to closed length of the clot increased, the removal pressures increased linearly from 0 to nearly 1000 mmHg, suggesting the largest correlation of reference states to EVT outcomes. Finally, as the length of the initial clot increased from 2 to 25 mm, the aspiration removal pressures increased in a seemingly logarithmic fashion, with clot length seeming to become less relevant as the clot's length increased beyond 10 mm. As the artery radii are not expected to vary significantly from person to person, the simulations with a clot lodged in an artery with increasingly smaller stress‐free inner radii was not performed.

## 4 Discussion

This study provides a framework for understanding the mechanics of embolization and lodging from an elastic standpoint. Most *in silico* work to date on the matter of EVT has focused on a stress‐free initial clot and artery whose reference configuration is also stress‐free [16, 32, 39, 40]. This is most likely not the case in AIS, as the kinetic energy required to transport a clot through the circulation (and opposed to gravity) is very quickly converted into elastic energy and internal stress. To date, there are conflicting reports suggesting that cardioembolic clots (i.e., clot origin) cause EVT complications, while other studies suggest hematocrit is the main factor at play [10, 15, 17, 18]. While these notions are not to be ignored, the kinematics of an elastic object lodging in a pre‐stressed artery need to be understood to make these determinations. To fully understand the limitations of current EVT techniques, a system that integrates the net energy of the system, as indicated by the initial stress state in the clot and the artery, needs to be taken into account. The data we present here suggest that a holistic approach to occlusive EVT simulations can provide a clearer picture of the complications that can arise in such a system.

**FIGURE 10:**
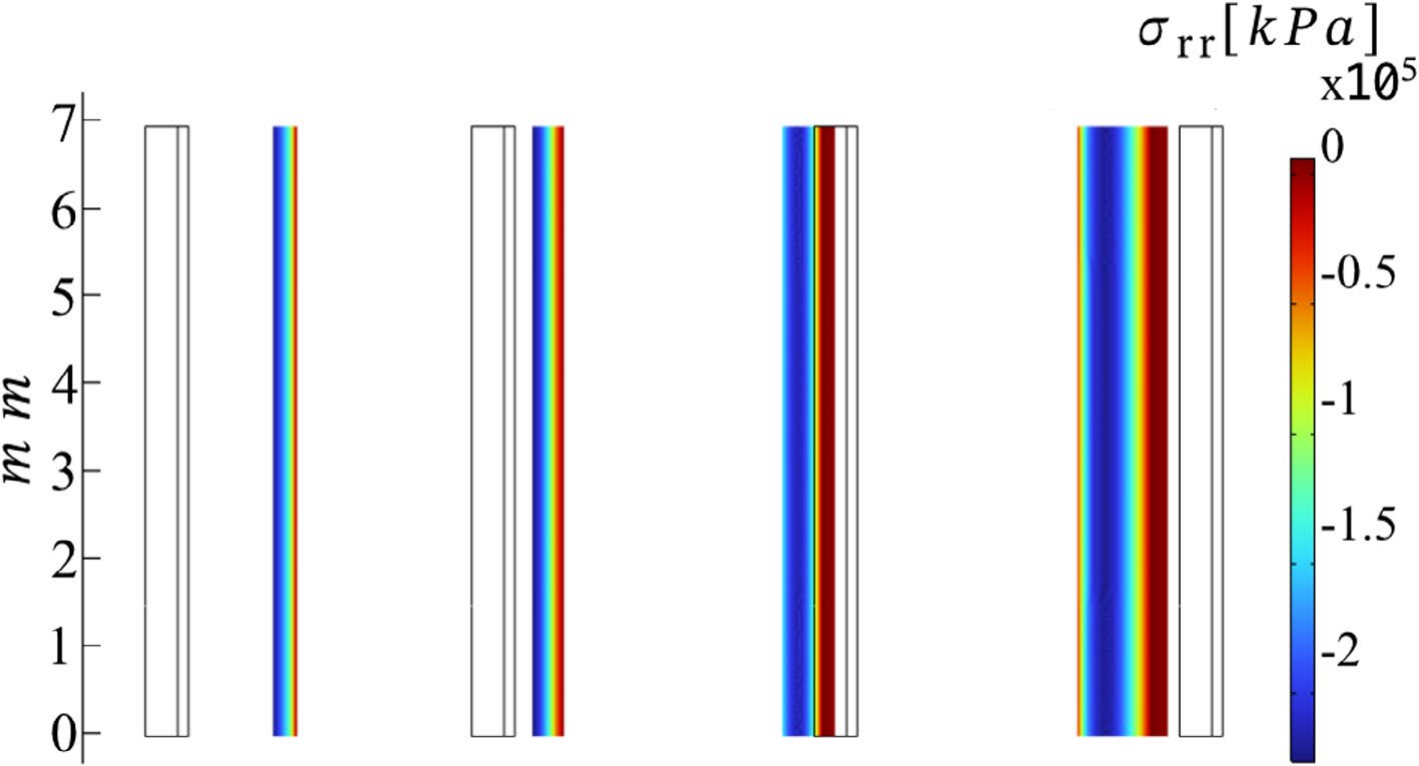
Successive initial, configurations of the media and adventitia wall, with stress‐free media inner radii are 0.4,0.65,0.95,1.25, showing the initial configuration relative to $\Omega_{i}^{\mathrm{cl}}$, the closed configuration. Outlines depict the closed‐configuration boundaries.

Arterial residual stress is a well‐known phenomenon, which increases the lifetime of vessel walls and decreases the stress gradient across the vessel wall [46, 77, 78]. The constitutive properties of cerebral vasculature are less well known, due to the sensitive relative anatomy involved. These data indicate that, even on the order of the M1 MCA, whose largest inner radius is nearly 3 mm, and whose wall thickness is less than 1 mm, initial stress states play a role in the outcomes of EVT. The opening angle, stress‐free inner radius, collagen fiber orientation, and even layer‐specific wall thicknesses are not known for these vessels. The interpolation used in this study assumes a similar structure in the M1 to other well‐known arteries, such as the common carotid. Assuming this to be the case, the reference radius of the artery is highly dependent on the opening angle, inner radius, and fiber orientation of the stress‐free artery. As the opening angle decreased, the vessel radius increased nearly 50%, and the wall thickness decreased by nearly the same percentage. Specifically, we found that as the artery opening angle increased, the equilibrium lumen diameter decreased (3.02 mm at $180^{{}^{\circ}}$ vs. 7.00 mm at $0^{{}^{\circ}}$), and the corresponding aspiration pressure required for the clot detachment increased. Thus, changes in the geometry under physiological pressure contribute directly to the observed relationship between opening angle and removal pressures. The inner radius of the stress‐free artery plays a significant role in the stress gradient across the arterial wall. Figure 10 shows successive decreases in inner wall stress‐free radius, with the compressive radial stresses increasing by 400%. Additionally, cerebral vasculature has structures, such as the glymphatic circulation, whose contribution to the mechanical behavior of these vessels is largely unknown [79]. For this reason, the actual residual stress state of the artery needs to be studied further, to provide base values for *in silico* work.

The stress state of the system becomes more complex with the addition of the clot into the closed domain. The artery, whose stress state was initially governed by its stress‐free dimensions, now deforms to accommodate a clot whose stress‐free dimensions add a measure of stored energy to the system. As a clot embolizes in the body, it will nearly always lodge in vasculature in such a way that the artery will deform radially outward, and in turn compress the clot inward. In works by previous groups, this aspect of the kinematics is not taken into account. In work by Oyekole et al. [39], Patki et al. [40] and Luraghi et al. [80], among others [81, 82], the clot was considered to be a right cylinder that conforms to the exact shape of the rigid vessel wall, when in all likelihood, the clot must be larger than the artery to fully lodge. The current study demonstrates how aspects like the clot stress‐free to closed‐configuration length affect the residual stress state of the system. A clot whose stress‐free radius is much larger than the closed domain will have nearly 40% higher compressive tractions into the arterial wall, adding that much additional force counteracting the retrieval devices. The same can be said for a clot whose shear modulus is orders of magnitude higher than those seen extracted from stroke patients [15], such as so‐called calcified cerebral emboli, which have mineral compositions reportedly commensurate with that of bone [83, 84, 85, 86]. While the material properties of the clot are well characterized, the mechanical behavior of the clot‐arterial interface is unknown. In work by our group, we have shown that clots exhibit adhesive tendencies on materials commonly used in medical devices, and it is thought that a similar interaction may occur on inflamed endothelial cells [28]. This type of interaction would add another element to this story. Overall, the mechanism of occlusion needs to be studied from a kinematic perspective, so that one can truly understand what forces need to be overcome to remove the blood clot from the vessel.

The aspiration part of the story allows us to glimpse into the behavior of the clot relative to current AIS treatment methods. Previously, studies have focused on the clot behavior relative to a rigid wall, as discussed in previous sections. This study indicates that the reactionary forces of the vessel onto the clot play a large role in the energy state of the system that needs to be overcome to remove the clot. Figure 9 shows this in stark detail, where aspects such as the stress‐free opening angle of the artery and the fiber orientation of the artery can increase the aspiration pressure necessary to remove the clot by upwards of 25% when compared to the base case. Aspiration pressures necessary to remove the clot can go up to nearly 900 mmHg when factors such as stress‐free to lodged length of the clot increase, suggesting clot burden plays a significant role in the outcomes of EVT. Anecdotally, several studies suggest that current aspiration thrombectomy techniques use mean aspiration pressures of roughly 400 mmHg, which are on the lower end of the spectrum needed to begin removal of the clot in most of these simulations. These data also contrast with previous studies, where the clot begins to detach from some distance between the distal and proximal clot‐artery interface, such as in Patki et al. [40], suggesting the addition of a pre‐stressed artery and a pre‐stressed clot changes the behavior of aspiration devices relative to the same stress‐free system. Overall, these data provide a spectrum of clot and arterial properties that can be used to compare to known variables from actual AIS clot retrievals to fill in more gaps in the knowledge base. This work emphasizes the need to understand the stress state of the cerebral arteries in relation to the clot types extracted to be able to better understand the energies that need to be overcome to remove all clot types.

Overall, this study provides a framework for implementing initial conditions in a reference geometry to simulate a system of a clot truly lodged in an artery with an inherited stress state. A clot that embolizes carries some level of energy that is then transferred into the occlusion site and stored locally in the cerebral arteries. The dimensions and properties of the clot before lodging are only part of the picture. If the clot lodges and migrates due to mean arterial pressure within the therapeutic window, the energy increases substantially as the ratio of the radius of the stress‐free clot to the inner radius of the artery increases. In this study, we provide a host of parameters that can be used as initial conditions for simulating this mechanical phenomenon, capturing the main aspects of the occlusion in a succinct equilibrium state that can be used to validate the efficacy of new EVT devices. This also emphasizes the need to consider the system holistically when determining routes of AIS treatment.

This study has several limitations that have been discussed in previous sections. Firstly, the residual stress state of the middle cerebral arteries is largely unknown. Cerebral arteries have glymphatic structures and fluid‐filled pockets around them that may dictate the internal stress state [79]. Concurrently, while literature reports show that human carotid arteries exhibit axial pre‐stretches closer to 20% [59], we chose a smaller pre‐stretch (1%) to isolate the effect of internally imposed arterial stresses rather than compounding effects. Future work will take into account the role of physiological axial stretch on clot detachment dynamics. Furthermore, clots are viscoelastic and heterogeneous and were considered to be elastic and homogeneous in this study. Due to the intricate nature of the problem, it became necessary to study equilibrium states of a homogeneous material, as the complex geometry and compounded system of pre‐deformations increased the computational cost of the calculations. We therefore elected to neglect the formation conditions and deformation history of the clot prior to lodging. Clearly, from a scientific viewpoint, this is a limitation of the study and there is ongoing work in our group to model an embolization process that results in a lodged thrombus. The piecewise manipulation of the parameters that govern the material response and residual stress state of the arteries is not without limitation. We note that in reality, if one were to modify the artery's opening angle, the overall model should be refit to the material behavior. In an attempt to characterize the singular effect of variations in one parameter at a time, we assumed a state of the system in which all other parameters were held constant. This was a purely modeling assumption and not reflective of actual changes in a physiological system. In fact, this study does not claim to be of immediate clinical significance; rather, it aims at offering a first framework for taking into consideration the residual stress states of the artery and clot in the context of acute ischemic stroke. The aspiration boundary condition was applied as a uniform negative pressure across the curved surface of the bulging clot in the initial configuration; however, this may not be the case clinically. If the catheter is not immediately in contact with the clot, the pressure boundary condition may vary across the surface of the clot until the catheter makes contact with the clot. At the point of contact, the pressure is assumed to act normal to the surface at all points, when in reality, the direction of the tractions is not known, and we therefore assume the boundary condition used here. The stress‐free and reference state of the clot are generally not known, and the idealized, right‐cylindrical geometries used here may not be fully representative of the shape of thromboemboli, and for this reason, idealized geometries were used. In order to enforce the axial strain that is common in arteries in vivo, and to suppress rigid body motion, roller boundary conditions were applied to the proximal and distal edges of the artery. While this may not be representative of the physiological system, it was an assumption made out of convenience for the enforcement of these conditions. Finally, the clot contact mechanics limited the outcomes of several simulations. In the case of the variation of clot reference length, at certain lengths, the top and bottom surfaces of the clot began to contact the lateral surface of the artery, leading to instabilities in the solution set. The contact problem between the clot free surface and the artery wall, as well as the clot‐artery interface, is a matter that will be incorporated into future studies.

## 5 Conclusions

This study provides a host of vessel and clot properties that can be used to determine the reference state in which thrombectomy devices encounter an occlusive thrombus. The data from this study suggest that both the artery and the clot determine the forces that need to be overcome to remove the thrombus. Aspects of the artery that impact this are the fiber orientation of collagen fibers, the stress‐free radius, and the opening angle. In the clot, the stress‐free dimensions and stiffness play a role in the necessary removal pressures, with clot burden having the highest impact. These data contrast previous studies and highlight the role of the initial state in the outcomes of clot removal procedures. The clinical implications of this are broad ranging, as the techniques currently in use seem to reach aspiration pressures commensurate with only beginning to dislodge the clot from the artery. Future aspiration techniques should take into consideration higher aspiration pressures and larger aspiration catheter radii. Overall, these data highlight the criticality of incorporating robust initial conditions for a clot lodged in an artery to truly evaluate the efficacy of current techniques and for the development of new methods.

## Ethics Statement

The authors have nothing to report.

## Conflicts of Interest

K.B.M. has a financial interest in Cranial Devices Inc., a company that could potentially benefit from the results of the presented research. The interest has been reviewed and is being managed by The Pennsylvania State University in accordance with its individual conflicts of interest policy, for the purpose of maintaining the objectivity of research at The Pennsylvania State University. The other authors declare no conflicts of interest.

## Supporting information


**Figure S1:** Supporting Information.

## Data Availability

The data that support the findings of this study are available from the corresponding author upon reasonable request.
