# Supplementary figures and images for: Characterization of the In Situ Stress State of Blood Clots in Ischemic Stroke: The Effect of Initial Conditions and Arterial Interaction
Source: Int J Numer Method Biomed Eng. 2025 Oct 3;41(10):e70094. doi: 10.1002/cnm.70094 (PMC12494024; doi:10.1002/cnm.70094)

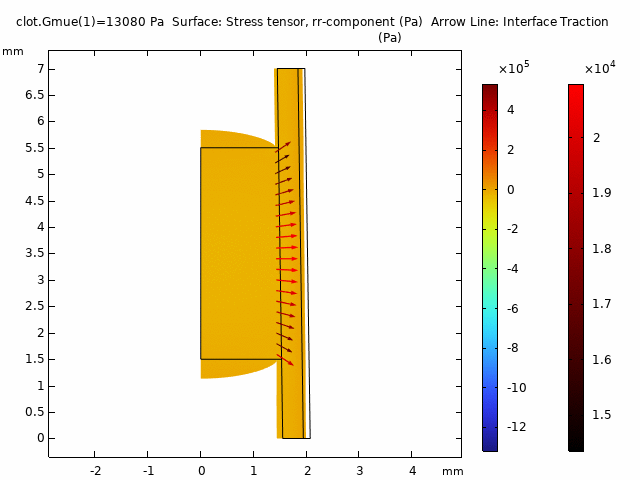

Supplement: Supplementary file 1 — Figure S1: Supporting Information. [file CNM-41-e70094-s001.gif]
